# Delay-induced uncertainty in the glucose-insulin system: Pathogenicity for obesity and type-2 diabetes mellitus

**DOI:** 10.3389/fphys.2022.936101

**Published:** 2022-09-01

**Authors:** Bhargav R. Karamched, George Hripcsak, Rudolph L. Leibel, David Albers, William Ott

**Affiliations:** ^1^ Department of Mathematics, Florida State University, Tallahassee, FL, United States; ^2^ Institute of Molecular Biophysics, Florida State University, Tallahassee, FL, United States; ^3^ Program in Neuroscience, Florida State University, Tallahassee, FL, United States; ^4^ Department of Biomedical Informatics, Columbia University, New York, NY, United States; ^5^ Division of Molecular Genetics, Department of Pediatrics, Vagelos College of Physicians and Surgeons, Columbia University Irving Medical Center, NY, NY, United States; ^6^ Naomi Berrie Diabetes Center, Columbia University Irving Medical Center, NY, NY, United States; ^7^ Section of Informatics and Data Science, Department of Pediatrics, Department of Biomedical Engineering, and Department of Biostatistics and Informatics, University of Colorado Anschutz Medical Campus, Aurora, CO, United States; ^8^ Department of Mathematics, University of Houston, Houston, TX, United States

**Keywords:** delay-induced uncertainty, glucostatic hypothesis, lyapunov exponent, obesity, shear, theory of rank-one maps, type-2 diabetes mellitus, ultradian model

## Abstract

We have recently shown that physiological delay can induce a novel form of sustained temporal chaos we call delay-induced uncertainty (DIU) (Karamched et al. (Chaos, 2021, 31, 023142)). This paper assesses the impact of DIU on the ability of the glucose-insulin system to maintain homeostasis when responding to the ingestion of meals. We address two questions. First, what is the nature of the DIU phenotype? That is, what physiological macrostates (as encoded by physiological parameters) allow for DIU onset? Second, how does DIU impact health? We find that the DIU phenotype is abundant in the space of intrinsic parameters for the Ultradian glucose-insulin model—a model that has been successfully used to predict glucose-insulin dynamics in humans. Configurations of intrinsic parameters that correspond to high characteristic glucose levels facilitate DIU onset. We argue that DIU is pathogenic for obesity and type-2 diabetes mellitus by linking the statistical profile of DIU to the glucostatic theory of hunger.

## 1 Introduction

Clinical and laboratory practice throughout biomedicine and biochemistry proceeds from the assumption that the dynamics of measured quantities are predictable. For instance, a clinician administers medication to a patient based on the supposition that the medical intervention will not induce an unexpectedly erratic response. The presence of sustained temporal chaos would fundamentally undermine the assumption of predictability. Such chaos has been observed in certain classical physiological models (Abarbanel et al. (1993); Li and Yorke (2004); Mackey and Glass (1977); Glass et al. (1988); Glass and Malta (1990)).

We recently proposed a novel route through which physiological delay can induce sustained temporal chaos for concrete dynamical systems of interest in biomedicine (Karamched et al. (2021)). We termed the resulting chaos *delay-induced uncertainty* (DIU). We argued that DIU is relevant for glycemic management in the intensive care unit by exhibiting it for the Ultradian model, an archetypal model of glucose-insulin dynamics (Sturis et al. (1991); Drozdov and Khanina (1995)). Tools from the general theory of nonuniformly hyperbolic dynamical systems and the theory of rank-one maps yielded a precise characterization of the dynamical and statistical profiles of DIU. Clinicians may find DIU difficult to interpret because these profiles can be subtle.

DIU is potentially relevant for any physiological system wherein delayed regulatory feedback controls try to maintain healthy homeostasis. Examples include pulmonary and respiratory dynamics (Mackey and Glass (1977); Sottile et al. (2018)), cardiac dynamics (Christini and Glass (2002)), female endocrine dynamics (Graham et al. (2020); Urteaga et al. (2019)), and neurological dynamics (Stroh et al. (2020); Claassen et al. (2013); Hodgkin and Huxley (1952)). Indeed, the use of mathematical physiology within medicine has broad potential (Albers et al. (2018a); Zenker et al. (2007)).

This paper is a first attempt to assess the impact of DIU on the ability of the glucose-insulin system to maintain homeostasis when responding to the ingestion of meals. We work with the Ultradian model as we did before (Karamched et al. (2021)), but in a different regime. Our previous work focused on glycemic management in the intensive care unit (ICU) and therefore considered the regime wherein the intrinsic (unforced) system admits a glycemic oscillation (limit cycle). Here, we work in the regime wherein the intrinsic system admits a stable stationary state. In this regime, meals (glucose kicks) move trajectories away from the stationary point. After each kick, the glucose-insulin control system tries to efficiently return to the fixed point. We are therefore interested in how DIU impacts *return to equilibrium*.

In the context of return to equilibrium, the recipe for DIU has three ingredients. First, delay renders the unforced system excitable by weakening the stability of the stationary point. Second, shear is present near this stationary point. One can think of shear as velocity gradients. Third, external forcing (glucose kicking) interacts with shear during the relaxation phase between kicks. This interaction stretches and folds the phase space, creating hyperbolicity in the dynamics and producing sustained temporal chaos.

Here, we show that the physiological architecture of the glucose-insulin system possesses all three ingredients in the DIU recipe. We offer substantial evidence for the following two conjectures.

1) The DIU phenotype is abundant in the space of intrinsic parameters. In other words, a variety of physiological macrostates (as encoded by intrinsic parameters) lead to DIU emergence.
2) DIU is pathogenic for obesity and type-2 diabetes mellitus (T2DM).

This paper is a call to action—a first step toward verifying these conjectures.

**FIGURE 1:**
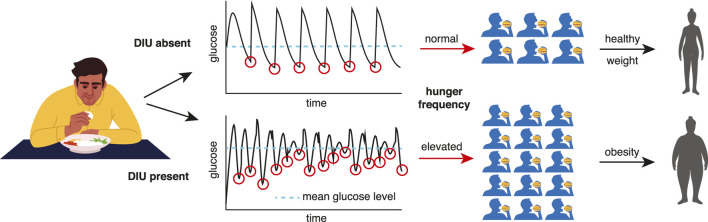
How DIU may be pathogenic for obesity and T2DM. When DIU is present, glucose level dips below its mean more frequently (bottom row). The glucostatic hypothesis asserts that such dips induce hunger. DIU would therefore impute elevated hunger frequency, leading to obesity on long timescales.

Given the importance of elucidating obesity pathogenesis (Schwartz et al. (2017)), the DIU pathogenicity conjecture is the primary contribution of this work. The two-part argument supporting it links the statistical distribution of glucose that DIU induces to the glucostatic theory (Chaput and Tremblay (2009); Mayer (1955)). First, when DIU is present, glucose level dips below its mean more frequently. Second, glucostatic theory asserts that such dips induce hunger. See Figure 1 for an illustration of this two-part argument. This conjectured form of obesity pathogenesis acts on long timescales (months and years). As we will show, DIU becomes more probable as intrinsic parameters move into regions of parameter space that correspond to elevated characteristic glucose levels. Development of early-stage obesity and T2DM would therefore act as a feedback mechanism by promoting DIU, leading to disease progression.

We work with the Ultradian model for two primary reasons, validity and flexibility. The model includes two major negative feedback loops describing effects of insulin on glucose use and glucose production. Both loops include glucose-based stimulation of insulin secretion. External forcing can include both meal ingestion and glucose infusion. The Ultradian model can be tuned so that the unforced system admits a limit cycle, as in (Karamched et al. (2021)), or a stationary state. Importantly, it has been used to accurately predict glucose dynamics in humans (Albers et al. (2017)).

## 2 The Ultradian model

In this section we describe the Ultradian glucose-insulin model (Sturis et al. (1991); Drozdov and Khanina (1995); Keener and Sneyd (1998)), the external forcing drive that we use for simulations, and intrinsic system parameters that we hypothesize can facilitate DIU onset.

**FIGURE 2:**
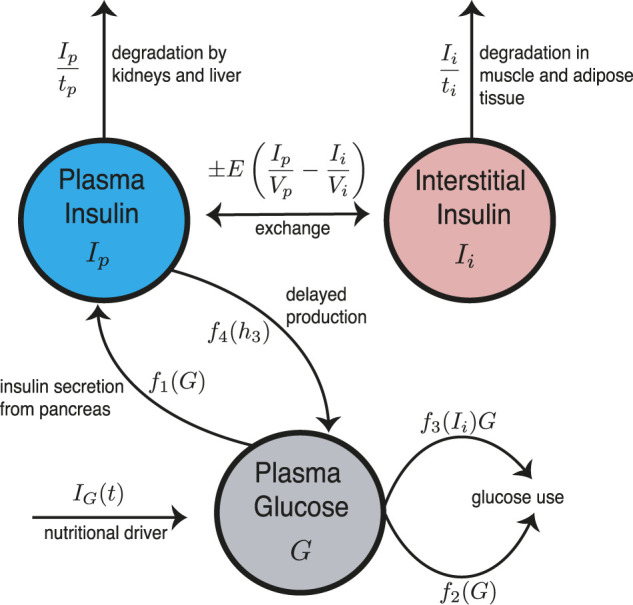
Schematic for the Ultradian model of glucose-insulin dynamics. Note the important delayed regulatory feedback between *I*_*p*_ and *G*.

The Ultradian model is a compartment model with three state variables: plasma glucose (*G*), plasma insulin (*I*_*p*_), and interstitial insulin (*I*_*i*_). See Figure 2 for the model schematic. These three state variables are coupled to a three-stage linear delay filter, producing a six-dimensional phase space. The model includes two major negative feedback loops describing effects of insulin on glucose use and glucose production. Both loops include glucose-based stimulation of insulin secretion. The Ultradian model includes physiologic delay, but the system is *finite-dimensional* because the delay assumes the form of a three-stage linear filter.

The full model is given by

$$\frac{dI_{p}}{dt}=f_{1}\left(G\right)-E\left(\frac{I_{p}}{V_{p}}-\frac{I_{i}}{V_{i}}\right)-\frac{I_{p}}{t_{p}}$$ (1a)

$$\frac{dI_{i}}{dt}=E\left(\frac{I_{p}}{V_{p}}-\frac{I_{i}}{V_{i}}\right)-\frac{I_{i}}{t_{i}}$$ (1b)

$$\frac{dG}{dt}=f_{4}\left(h_{3}\right)+I_{G}\left(t\right)-f_{2}\left(G\right)-f_{3}\left(I_{i}\right)G$$ (1c)

$$\frac{dh_{1}}{dt}=\frac{1}{t_{d}}\left(I_{p}-h_{1}\right)$$ (1d)

$$\frac{dh_{2}}{dt}=\frac{1}{t_{d}}\left(h_{1}-h_{2}\right)$$ (1e)

$$\frac{dh_{3}}{dt}=\frac{1}{t_{d}}\left(h_{2}-h_{3}\right),$$ (1f)

where *f*_1_(*G*) represents the rate of insulin production, *f*_2_(*G*) represents insulin-independent glucose use, *f*_3_(*I*_*i*_)*G* represents insulin-dependent glucose use, and *f*_4_ (*h*_3_) represents delayed insulin-dependent glucose use. The functional forms of *f*_1_, *f*_2_, *f*_3_, and *f*_4_ are given by

$$f_{1}\left(G\right)=\frac{R_{m}}{1+\exp\left(\frac{-G}{V_{g}C_{1}}+a_{1}\right)}$$ (2a)

$$f_{2}\left(G\right)=U_{b}\left(1-\exp\left(\frac{-G}{C_{2}V_{g}}\right)\right)$$ (2b)

$$f_{3}\left(I_{i}\right)=\frac{1}{C_{3}V_{g}}\left(U_{0}+\frac{U_{m}-U_{0}}{1+{\left(\kappa I_{i}\right)}^{-\beta }}\right)$$ (2c)

$$f_{4}\left(h_{3}\right)=\frac{R_{g}}{1+\exp\left(\alpha \left(\frac{h_{3}}{C_{5}V_{p}}-1\right)\right)},$$ (2d)

with

$$\kappa =\frac{1}{C_{4}}\left(\frac{1}{V_{i}}-\frac{1}{Et_{i}}\right).$$ (3)

Table 1 summarizes the meaning of each model parameter and provides the set of nominal parameter values.

**TABLE 1:** Full list of intrinsic parameters for the Ultradian glucose-insulin model (Albers et al. (2017)). Note that IIGU and IDGU denote insulin-independent glucose utilization and insulin-dependent glucose utilization, respectively.

Ultradian model parameters		
Name	Nominal value	Meaning
*V*_*p*_	3 L	plasma volume
*V*_*i*_	11 L	interstitial volume
*V*_*g*_	10 L	glucose space
*E*	0.2 L min^−1^	exchange rate for insulin between remote and plasma compartments
*t*_*p*_	6 min	time constant for plasma insulin degradation (via kidney and liver filtering)
*t*_*i*_	100 min	time constant for remote insulin degradation (via muscle and adipose tissue)
*t*_*d*_	10.5 min	delay between plasma insulin and glucose production
*R*_*m*_	209 mU min^−1^	linear constant affecting insulin secretion
*a*_1_	6.6	exponential constant affecting insulin secretion
*C*_1_	300 mg L^−1^	exponential constant affecting insulin secretion
*C*_2_	144 mg L^−1^	exponential constant affecting IIGU
*C*_3_	100 mg L^−1^	linear constant affecting IDGU
*C*_4_	80 mU L^−1^	factor affecting IDGU
*C*_5_	26 mU L^−1^	exponential constant affecting IDGU
*U*_*b*_	72 mg min^−1^	linear constant affecting IIGU
*U*_0_	4 mg min^−1^	linear constant affecting IDGU
*U*_*m*_	94 mg min^−1^	linear constant affecting IDGU
*R*_*g*_	180 mg min^−1^	linear constant affecting IDGU
*α*	7.5	exponential constant affecting IDGU
*β*	1.772	exponent affecting IDGU

### 2.1 Pulsatile glucose forcing drives

The term *I*_*G*_(*t*) in Eq.1c represents the external nutritional drive. We call system (1) without this term the *intrinsic system* or *unforced system*. In this paper, we consider an idealized nutritional drive *I*_*G*_(*t*) that consists of pulsatile kicks. This drive models meals that are eaten and digested instantaneously. That is, we assume that the nutritional content of each meal immediately affects the glucose state variable in the Ultradian system. The idealized nutritional drive is given by

$$I_{G}\left(t\right)=\overset{\infty }{\underset{n=1}{\sum }}A_{n}\delta \left(t-T_{n}\right),$$ (4)

where *δ*(*t*) is the Dirac delta distribution (unit impulse), *T*_*n*_ is the time of meal *n*, and *A*_*n*_ is the amount of carbohydrate in meal *n*. Importantly, this pulsatile drive does not overwhelm the intrinsic dynamics. On the contrary, it can interact subtly with intrinsic shear to produce DIU, as we will see.

The form of *I*_*G*_(*t*) in Eq. 4 induces the following dynamics. Between two consecutive kicks (*T*_*n*−1_ < *t* < *T*_*n*_), Ultradian dynamics evolve according to system (1) with *I*_*G*_(*t*) = 0. At time *T*_*n*_ of meal *n*, the glucose state variable, *G*, undergoes the instantaneous change *G*↦*G* + *A*_*n*_. That is, at time *T*_*n*_ we pause the flow generated by the intrinsic system and apply the diffeomorphism

$$\left(I_{p},I_{i},G,h_{1},h_{2},h_{3}\right)\mapsto \left(I_{p},I_{i},G+A_{n},h_{1},h_{2},h_{3}\right)$$ (5)

to the phase space. We call this diffeomorphism followed by flow of the intrinsic system cycle the *kick-relaxation cycle*.

In reality, meals produce glucose perturbations that are temporally localized but not instantaneous. Nevertheless, we have strong evidence that the emergence of DIU (or the absence of such emergence) is sensitive to neither the exact timing of the pulses nor to their shape. In previous work (Karamched et al. (2021)), we examined the emergence of DIU for the Ultradian model when the delay parameter *t*_*d*_ is tuned so that the intrinsic system admits a limit cycle (sustained oscillatory dynamics). There, we showed that DIU can emerge when each inter-meal time is drawn from an exponential distribution (Poissonian inter-meal timing) and when the drive 4) is replaced with square pulses of duration 30 min that arrive at 8 a.m., noon, and 6 PM. Here, we elect to work with drive 4) and consider only periodic pulsing (*T*_*n*_ = *nT*, where $T\in R_{>0}$ is the inter-kick time) with constant kick amplitude (*A*_*n*_ = *A* for all $n\in Z_{⩾0}$ ) in order to focus on how intrinsic parameters impact DIU emergence. Our previous work indicates that our new results for periodic pulsatile forcing will continue to hold for more complex forcing drives.

### 2.2 Key intrinsic parameters for DIU emergence

We hypothesize that intrinsic (unrelated to the forcing drive) parameters directly linked to *G*, the glucose state variable, play a key role in DIU onset. This hypothesis is partially inspired by recent work that established a positive correlation between mean glucose levels and glucose variance (Albers et al. (2018b)). Our numerical experiments examine the impact of the following parameters on DIU emergence.

• *R*_*g*_ - the uninhibited hepatic glucose production rate
• *U*_*b*_ - the maximal insulin-independent glucose usage rate
• *U*_0_ - the basal insulin-dependent glucose usage rate
• *α* - the inhibition of hepatic glucose production
• *a*_1_ - the basal glucose-based insulin inhibition
• *C*_1_ - the sensitivity of insulin production to glucose

Importantly, each of these intrinsic parameters has a concrete physiological interpretation.

## 3 Methods

**The maximal Lyapunov exponent as a diagnostic tool.** We use the maximal Lyapunov exponent, Λ_max_, as a DIU diagnostic: Λ_max_ > 0 indicates DIU whereas Λ_max_ < 0 indicates its absence. Computing Λ_max_ requires solving system (1). We do this in the following way. During the relaxation intervals (*T*_*n*−1_, *T*_*n*_) between kicks, we integrate the unforced differential equations using the MATLAB ode23s solver. At kick times *T*_*n*_, we pause the differential equation solver and apply the diffeomorphism of phase space induced by the kick (see Eq. 5).

We compute the maximal Lyapunov exponent in the following way. We track two solutions to system (1), initially separated by *d*_0_ = 10^–8^. One of these solutions can be thought of as a base solution and the other as a perturbation. After the first kick-relaxation cycle, we compute the separation *d*_1_ between the solutions and store the quantity log (*d*_1_/*d*_0_) in a vector. We then renormalize by rescaling the secondary orbit so that the distance between the solutions resets to *d*_0_. We proceed in this manner for 10^5^ kick-relaxation cycles. This produces a vector containing 10^5^ values of log (*d*_1_/*d*_0_). Averaging over the vector produces Λ_max_. The maximal Lyapunov exponent consequently quantifies the amount of expansion per kick-relaxation cycle.

## 4 Results

We have designed our numerical experiments to support two primary conjectures. These conjectures are the animating force behind this paper.

1) The DIU phenotype is abundant in the space of intrinsic parameters. In other words, a variety of physiological macrostates (as encoded by intrinsic parameters) lead to DIU emergence.
2) DIU is pathogenic for obesity and T2DM.

### 4.1 Numerical experiments: Design, rationale, and expectations

**Tuning of intrinsic parameters.** To support the conjecture that the DIU phenotype is abundant in the space of intrinsic parameters, we begin by setting the intrinsic parameters in the unforced Ultradian model to the nominal values listed in Table 1. Crucially, the delay timescale *t*_*d*_ acts as a bifurcation parameter for the intrinsic system. There exists a value $t_{d}^{*}$ at which the intrinsic system undergoes a supercritical Hopf bifurcation. The intrinsic system admits a stable stationary point (*I*_*p*,eq_, *I*_*i*,eq_, *G*_eq_, *h*_1,eq_, *h*_2,eq_, *h*_3,eq_) for $t_{d}<t_{d}^{*}$ (homeostasis) that gives birth to a stable limit cycle (glycemic oscillation) for $t_{d}>t_{d}^{*}$ . For our numerical experiments, we set *t*_*d*_ to the nominal value 10.5 min, a value strictly less than $t_{d}^{*}$ , thereby placing the intrinsic system in the stable stationary point regime. This is the appropriate regime for our current study because we are interested in how the dynamical variables relax to homeostatic levels between glucose kicks.

Using the nominal values of the intrinsic parameters as a starting point, we look for DIU along six one-dimensional slices of parameter space. We select a parameter from the list given in Section 2.2 and then vary this parameter while holding all other intrinsic parameters fixed.

**Testing for DIU onset.** Having set the intrinsic parameters, we test for DIU onset by tuning the external pulsatile forcing drive (4). For the sake of simplicity, we select a kick amplitude *A* and set *A*_*n*_ = *A* for all $n\in Z_{⩾0}$ . We work with periodic kicks, so we set *T*_*n*_ = *nT*, where *T* is the time between consecutive kicks. The forcing drive 4) for the experiments is therefore given by

$$I_{G}\left(t\right)=A\overset{\infty }{\underset{n=1}{\sum }}\delta \left(t-nT\right).$$ (6)

To test for DIU onset, we compute the maximal Lyapunov exponent Λ_max_ as a function of *T*.

**Expectations.** DIU may or may not emerge as *T* increases, depending on the dynamics of the intrinsic flow near the stationary point. If contraction to the stationary state is strong and shear near the stationary state is weak, DIU will not emerge. The maximal Lyapunov exponent Λ_max_ will indicate this by remaining negative as *T* increases. In fact, Λ_max_ will *decrease* as *T* increases because the phase space has more time to contract between kicks as *T* increases.

On the other hand, if contraction to the stationary state is weak and shear near the stationary state is strong, then DIU can emerge as *T* increases. This can happen because when *T* is large, shear has a long time to act between kicks. Shear causes the phase space to stretch and fold, thereby producing DIU. In our experiments, a transition from Λ_max_ < 0 to Λ_max_ > 0 as *T* increases indicates that DIU has emerged.

### 4.2 The DIU phenotype is abundant in the space of intrinsic parameters

**FIGURE 3:**
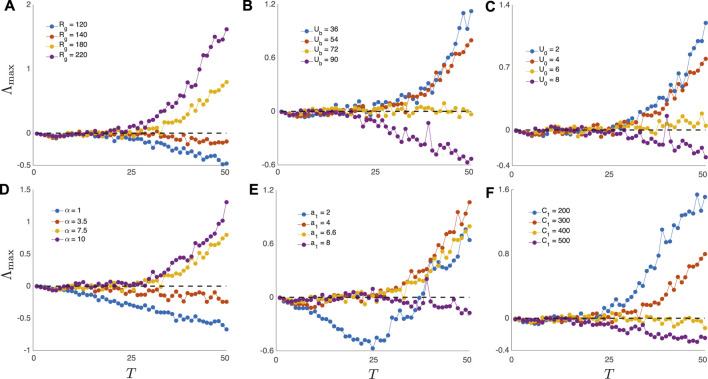
The DIU phenotype is abundant in the space of intrinsic parameters. Plots show the maximal Lyapunov exponent Λ_max_ as a function of inter-kick time *T* for the time-*T* map induced by the Ultradian system (1) with *T*-periodic pulsatile forcing (6). DIU is present when Λ_max_ > 0 and absent when Λ_max_ < 0. As *T* increases, DIU emerges when intrinsic parameters are tuned so as to increase characteristic glucose levels. Intrinsic parameters are set to the nominal values in Table 1 except for the single intrinsic parameter that is tuned in each panel: **(A)** *R*_*g*_; **(B)** *U*_*b*_; **(C)** *U*_0_; **(D)** *α*; **(E)** *a*_1_; **(F)** *C*_1_. Kick amplitude: *A* = 10 mg/dl.

Figure 3 illustrates how Λ_max_ varies with *T* as we individually tune each of the six parameters identified in Section 2.2. Each panel corresponds to tuning a single parameter while holding all other intrinsic parameters fixed at the nominal values. Importantly, DIU emerges in every one of the six panels when we tune the selected parameter so as to increase characteristic glucose levels in the intrinsic dynamics.

**FIGURE 4:**
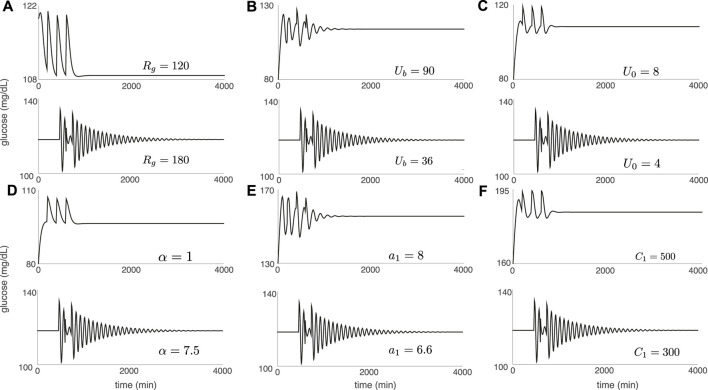
Glucose trajectories generated by the Ultradian system (1) with forcing that consists of three glucose kicks spaced 100 min apart. After the final kick, the glucose level converges to the equilibrium value *G*_eq_. Convergence is either rapid (top of each panel) or via a slow damped oscillation (bottom of each panel). Intrinsic parameters are set to the nominal values in Table 1 except for the single intrinsic parameter that is tuned in each panel: **(A)** *R*_*g*_; **(B)** *U*_*b*_; **(C)** *U*_0_; **(D)** *α*; **(E)** *a*_1_; **(F)** *C*_1_. Kick amplitude: *A* = 10 mg/dl.

Figure 4 confirms the expected link between strength of contraction to the stationary point, shear near the stationary point, and DIU emergence. For Figure 4, we replace the periodic pulsatile forcing used to generate Figure 3 with a forcing signal that consists of three kicks (meals). After the final kick, the glucose variable converges to the equilibrium level *G*_eq_ as *t* → *∞*. The panels in Figure 4 indicate that our experiments have captured two behaviors. Either we see rapid convergence to *G*_eq_ (as in Figure 4D (top)), or we see slow convergence to *G*_eq_ by way of a damped oscillation (as in Figure 4D (bottom)). Notice that in each panel of Figure 4, we tune the same parameter that we tune in the corresponding panel of Figure 3, while holding all other intrinsic parameters fixed at the nominal values.

Comparing Figures 3, 4 shows that without exception, *the geometry of the glucose trajectory predicts whether or not DIU will emerge*. If we observe rapid convergence to *G*_eq_, as in Figure 4D (top) for instance, then DIU does not emerge. If, however, we observe slow convergence to *G*_eq_ by way of a damped oscillation, as in Figure 4D (bottom) for instance, then DIU emerges.

**FIGURE 5:**
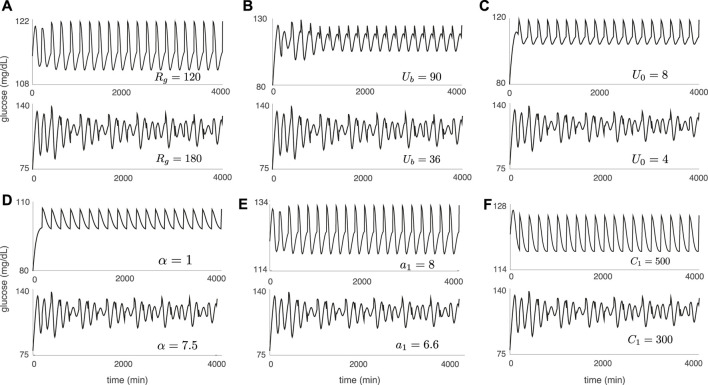
Sustained temporal chaos associated with DIU. Plots show glucose trajectories produced by the Ultradian system (1) with *T*-periodic pulsatile forcing (6). Each trajectory in Figure 5 corresponds to a companion trajectory in Figure 4. When DIU is absent, *T*-periodic pulsatile forcing results in a rhythmic glucose signal (top of each panel). When DIU is present, we observe sustained temporal chaos (bottom of each panel). Intrinsic parameters are set to the nominal values in Table 1 except for the single intrinsic parameter that is tuned in each panel: **(A)** *R*_*g*_; **(B)** *U*_*b*_; **(C)** *U*_0_; **(D)** *α*; **(E)** *a*_1_; **(F)** *C*_1_. Forcing parameters: *A* = 10 mg/dl, *T* = 100 min.

Figure 5 illustrates the DIU dynamical profile and acts as a companion to Figure 4. Each glucose trajectory in Figure 5 results from forcing with periodic pulsatile kicks 6) and corresponds to a companion glucose trajectory in Figure 4 (produced by applying only three kicks). When contraction toward the equilibrium glucose level *G*_eq_ is strong (Figures 4A–F (top)), driving with periodic pulsatile kicks produces rhythmic behavior (Figures 5A–F (top)). When periodic pulsatile kicks produce DIU, glucose trajectories exhibit sustained temporal chaos (Figures 5A–F (bottom)).

### 4.3 DIU is pathogenic for obesity and T2DM

We have established that the DIU phenotype is abundant in the space of intrinsic parameters for the Ultradian model. But why does this matter? Delayed regulatory feedback pathways are common in mathematical physiology. Since DIU emerges in a natural way for the Ultradian model, it may appear in a variety of physiological models. When present, DIU can profoundly impact medical practice because medicine proceeds from the assumption that the outcome of an intervention can be predicted when the state of the patient at the time of intervention is known. Sustained temporal chaos undercuts this assumption. See (Karamched et al. (2021)) for an assessment of the impact of DIU on glycemic management in the intensive care unit.

Here, we conjecture that DIU is pathogenic for obesity and T2DM. This conjecture is based on how the statistical signature of DIU links to the glucostatic theory. The glucostatic theory asserts that drops in blood glucose levels induce hunger and therefore energy intake (Chaput and Tremblay (2009); Mayer (1955)). If such drops are frequent in time and sizable in magnitude, excess energy intake could result.

**FIGURE 6:**
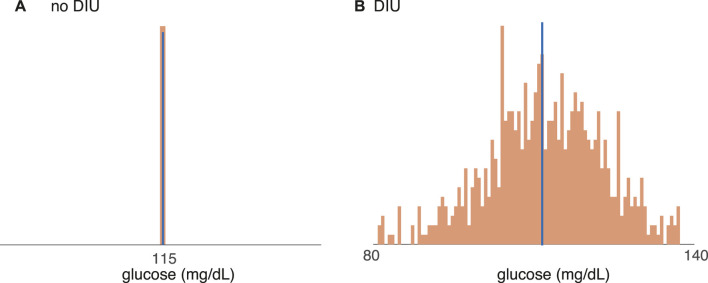
Support for the conjecture that DIU is pathogenic for obesity and T2DM. Distributions of the glucose variable (7) for the time-*T* map induced by the Ultradian system (1) with *T*-periodic pulsatile forcing (6). Blue bar indicates mean. **(A)** When *R*_*g*_ = 120 mg/min, DIU is absent and the glucose distribution concentrates at the mean. **(B)** When *R*_*g*_ = 180 mg/min, DIU is present. Consistent with the mathematical structure of the DIU profile, the glucose distribution is approximately Gaussian. All of the other intrinsic parameters are set to the nominal values in Table 1. Forcing parameters: *A* = 10 mg/dl, *T* = 100 min.

Figure 6 shows that DIU induces frequent, sizable drops in blood glucose levels! We start with all intrinsic parameters set at the nominal values and we then tune *R*_*g*_, the uninhibited hepatic glucose production rate. Figure 6 shows glucose distributions for the time-*T* map induced by Ultradian dynamics 1) with *T*-periodic pulsatile forcing (6). That is, each histogram gives the distribution of

$$\left\{G\left(nT\right):n\in Z_{⩾0}\right\}$$ (7)

for a different value of *R*_*g*_. We set *T* = 100 min. When *R*_*g*_ = 120 mg/min (Figure 6A), a value for which DIU is absent, the glucose distribution is essentially a Dirac measure concentrated at the mean. (Blue indicates the mean of the glucose distribution and orange indicates the distribution itself.) However, when *R*_*g*_ = 180 mg/min (Figure 6B), a value for which DIU is present, the glucose distribution is approximately Gaussian. This is as it should be—the mathematical theory behind DIU predicts Gaussian statistics when DIU is present. Notice that the variance of the approximately Gaussian distribution is large. This means that the glucose level frequently drops well below its mean. In light of glucostatic theory, this observation directly supports the conjecture that DIU is pathogenic for obesity and T2DM.

### 4.4 DIU emerges for generic pulsatile meal drives

Our results do not depend on the precise form of the pulsatile forcing that appears in (6). The forcing need not be periodic, and it need not consist of *δ*-pulses. DIU should emerge for a generic pulsatile forcing drive as long as the forcing interacts with intrinsic shear^1^.

**FIGURE 7:**
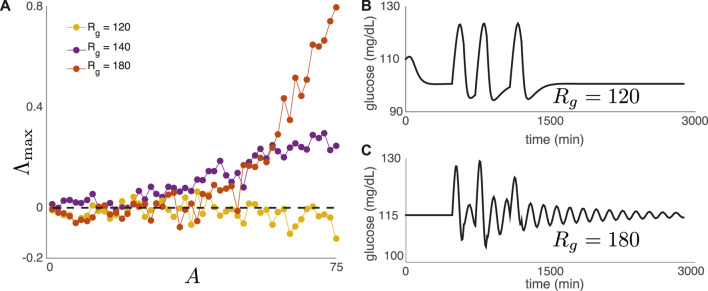
DIU phenotype for a realistic nutritional driver. **(A)** We have replaced (6) with the exponential-type drive in (8). Meals are consumed daily at 8 a.m., noon, and 6 p.m. Plot shows Λ_max_ as a function of meal amplitude *A* for three values of *R*_*g*_ **(B,C)** We replace (8) with a single day of meals (three meals). Rapid return to equilibrium correlates with the absence of DIU, while slow, oscillatory return to equilibrium correlates with the presence of DIU. Here, *A* = 50 mg/(dL ⋅ min) and *υ* = 1/120 min^−1^.

To support this claim, we have varied the intrinsic parameter *R*_*g*_ to test for DIU emergence after replacing 6) with

$$I_{G}\left(t\right)=A\overset{\infty }{\underset{n=1}{\sum }}\Theta \left(t-m_{n}\right)e^{-\upsilon \left(t-m_{n}\right)},$$ (8)

where *A* > 0 denotes meal amplitude, Θ(*t*) is the Heaviside function, *υ* > 0 is a constant, and *m*_*n*_ denotes the time of meal *n*. For this set of experiments, meals are consumed daily at 8 a.m., noon, and 6 p.m. Figure 7A shows Λ_max_ as a function of meal amplitude *A* for three values of *R*_*g*_. For two of the three values of *R*_*g*_, the top Lyapunov exponent becomes positive as *A* increases, indicating DIU onset. For Figure 7bc, we replace (8) with a single day of meals (three meals) in order to show that the nature of return to equilibrium correlates with DIU onset. Rapid return to equilibrium correlates with the absence of DIU (Figure 7B), while slow, oscillatory return to equilibrium correlates with the presence of DIU (Figure 7C).

## 5 Discussion

We have found that DIU is abundant in the space of parameters for the Ultradian glucose-insulin model. Such DIU could result in obesity and T2DM if induced low-glucose excursions produce excess hunger frequently enough, but much work remains to verify the conjecture that DIU is pathogenic for obesity and T2DM. Crucially, DIU and the theory behind it must be anchored to data. Methods for DIU detection directly from data should be developed for the clinical and self-care settings. The impact of DIU on the techniques by which models are fit to data should be assessed.

We have assumed in this paper that the intrinsic parameters in the Ultradian model do not vary over time. On long timescales, however, DIU may affect physiological state. At the modeling level, this would correspond to DIU causing intrinsic model parameters to drift (perhaps slowly) over time. Such drift might enhance the pathogenicity of DIU through a feedback mechanism: When DIU is present, intrinsic parameters may slowly drift into a region of parameter space that is even more favorable for DIU. A mathematical investigation of this phenomenon would involve developing a theory of DIU for nonstationary dynamical systems.

We have shown here that the DIU phenotype is abundant in the space of intrinsic parameters for the Ultradian model. An important next step will be to precisely characterize the DIU phenotype in terms of physiological architecture. Such a characterization may reveal the most essential physiological mechanisms that lead to DIU onset. Mathematically speaking, we must quantify shear near stationary states of flows. Shear near limit cycles has received considerable attention (Ott and Stenlund (2010); Wang and Young (2003)). Shear near stationary states, though, has only been quantified in dimension two (Ott (2008)).

The rigorous mathematical theory behind DIU is known as the theory of rank-one maps. This theory has been developed for finite-dimensional dynamical systems (Wang and Young (2001, 2008; 2013)). The Utradian model is finite-dimensional as a dynamical system because the delay in the Ultradian model takes the form of a three-stage linear filter. The theory of rank-one maps therefore characterizes the sustained temporal chaos that we see in the Ultradian model. However, models that include explicit delays—systems of nonlinear delay differential equations—permeate mathematical physiology. Models that include explicit delays are infinite-dimensional when viewed as dynamical systems. Important infinite-dimensional analogs of the Ultradian model have been studied (Li et al. (2006); Li and Kuang (2007)). The theory of rank-one maps must be extended to infinite-dimensional dynamical systems in order to analyze delay differential equations in the DIU context. See (Lu et al. (2013)) for an approach that combines the existing theory of rank-one maps with invariant manifold techniques.

When assessing the impact of DIU on a given physiological system, one should ask the following questions. Are we interested in precisely predicting the temporal evolution of individual orbits, or do we care more about the statistics of the system? What are the relevant timescales? For the glucose-insulin system, we have now studied two contrasting settings. In the ICU context, we showed that DIU can disrupt single-orbit prediction on short timescales (Karamched et al. (2021)). In the present paper, we have argued that over long timescales, DIU-induced glucose statistics may be pathogenic for obesity and T2DM.

## Data Availability

The original contributions presented in the study are included in the article/supplementary materials, further inquiries can be directed to the corresponding author/s. The code used to generate all figures is available here: https://github.com/Bargo727/DIU.

## Footnotes

1 Here we compute Λ_max_ by averaging over time intervals of length 12 h.
